# Cryopreserved Mucosal Olfactory Ensheathing Cells Promote Functional Recovery After Dorsal Root Injury

**DOI:** 10.3390/cells15100944

**Published:** 2026-05-20

**Authors:** Kamile Minkelyte, Daqing Li, Ying Li, Ahmed Ibrahim

**Affiliations:** 1 Spinal Repair Unit, Department of Translational Neuroscience and Stroke, UCL Queen Square Institute of Neurology, University College London, Queen Square, London WC1N 3BG, UK; daqing.li@ucl.ac.uk (D.L.); ying.li@ucl.ac.uk (Y.L.); ahmed.ibrahim@ucl.ac.uk (A.I.); 2 Imperial College Healthcare NHS Trust, London W2 1NY, UK

**Keywords:** olfactory ensheathing cells, spinal cord injury, transplantation, neuroregeneration, cryopreservation

## Abstract

Olfactory ensheathing cell (OEC) transplantation has been widely shown to support axonal regeneration, remyelination, and functional recovery after central nervous system injury; however, autologous approaches are limited by low cell yields from patient biopsies, which may be insufficient for large spinal cord lesions. This study evaluated whether cryopreservation could provide a scalable alternative by preserving the therapeutic potential of mucosa-derived OECs. Using a rat dorsal root injury model, cryopreserved mucosa-derived OECs (CmOECs) were thawed and assessed for viability, phenotype, and efficacy following transplantation. Although total viable cell yield was reduced compared with primary cultures, the relative proportion of OECs remained stable, and cells retained characteristic morphology and marker expression in vitro. In vivo, transplantation of CmOECs resulted in significant functional recovery in climbing and forepaw fault tasks compared with injured controls, with outcomes comparable to primary mucosal OEC transplantation. Immunohistochemical analysis confirmed the survival and integration of transplanted cells at the dorsal root entry zone, alongside evidence of axonal regeneration and astrocytic remodeling. These findings demonstrate that mucosa-derived OECs retain therapeutic efficacy following cryopreservation and support the development of standardized OEC biobanks as a scalable strategy for spinal cord repair.

## 1. Introduction

Spinal cord injury (SCI) is a devastating condition that results in partial or complete loss of motor, sensory, and autonomic functions. Despite advances in supportive care and rehabilitation, there are currently no effective treatments capable of reversing the underlying nerve damage or restoring neurological function after SCI [1,2,3]. Among the various experimental therapeutic strategies under investigation, cell transplantation has emerged as one of the most promising approaches due to its potential to repair damaged neural tissue, promote axonal regeneration, and restore neurological function [4,5].

Olfactory ensheathing cells (OECs), a unique population of glial cells found within the olfactory system, have attracted significant interest due to their natural role in guiding the continual regeneration of olfactory axons throughout life [6,7,8,9,10]. These cells can be harvested from two primary sources: the olfactory bulb (bOECs) and the olfactory mucosa (mOECs). OECs derived from the olfactory bulb have shown regenerative properties in both preclinical models and in clinical trials [9,11,12,13,14,15,16]. However, obtaining bOECs typically requires highly invasive procedures such as transnasal endoscopic or transcranial biopsies, which carry considerable risks and are clinically impractical [17]. Moreover, recent studies have shown that SCI initiates degenerative changes within the brain, including the olfactory bulb, leading to dysregulation and impaired neurogenesis [18,19]. These pathological changes may compromise the viability and regenerative capacity of harvested bOECs, resulting in unpredictable yields and an increased risk of culture failure.

An alternative and clinically more accessible source of OECs is the olfactory mucosa, which can be obtained through minimally invasive nasal biopsies [20,21,22]. Although mOECs typically yield fewer cells per sample than bOECs, our previous work demonstrated that supplementing culture media with N2, insulin–transferrin–selenium (ITS), or forskolin can significantly enhance OEC yield [23]. Cryopreservation offers an additional strategy to overcome the limitation of low cell numbers by enabling the pooling of multiple autologous or allogeneic samples over time, thereby generating sufficient quantities for transplantation.

Using autologous mOECs offers several advantages: cells are immunologically compatible, thereby eliminating the need for immunosuppressive therapy and reducing the risk of rejection. Alternatively, the use of allogeneic samples would allow for the creation of OEC biobanks, providing an on-demand source of cells that could shorten the interval between injury and transplantation, reduce the effects of secondary injury [24,25,26,27], and expand applicability to both acute and chronic cases. Allografts carry an inherent risk of immune rejection; however, in our earlier work, xenogeneic transplantation of mouse-derived bulbar OECs into rats with chronic corticospinal tract injuries, combined with daily cyclosporine administration, led to recovery of goal-directed forepaw reaching. Notably, after this functional improvement was established, immunosuppression was discontinued. Despite subsequent loss of the transplanted cells due to rejection, the recovered function persisted. These findings suggest that the primary role of the grafted cells is to facilitate axonal bridging across the lesion, and that their long-term survival is not required to sustain the functional gains [28].

Compared to bOECs, mOECs retain many of the essential regenerative properties, including secretion of neurotrophic factors, guidance of axonal growth, and modulation of the injury environment. Additionally, there is evidence that these cells may be more beneficial compared to bOECs. Mucosal OECs have been shown to have increased capacity for migration, cavity prevention, and axonal growth in spinal cord injury rat models [29,30]. A growing number of research groups are focusing on mOECs due to their accessibility and therapeutic promise.

Our recent research demonstrated that transplantation of cryopreserved bOECs could promote axonal regeneration and improve functional recovery in an experimental rat model [31]. Building on these findings, the current study investigates whether cryopreserved mOECs exhibit similar regenerative and therapeutic potential.

## 2. Materials and Methods

All animal procedures were conducted in accordance with the UK Home Office regulations for the care and use of laboratory animals, as outlined in the UK Animals (Scientific Procedures) Act 1986, and were approved by the ethical committee of the University College London (UCL) Institute of Neurology. Adult female Sprague–Dawley (SD) rats (150–200 g) were obtained from Charles River, UK. Additionally, adult green rats, provided as a gift by the UCL School of Pharmacy, were used as the source of transplant cells.

### 2.1. Experimental Design

Control Group: Animals underwent unilateral dorsal root transection at C6–T1 and did not receive any cell transplantation (*n* = 6).

Transplant Groups: Three experimental groups were included:

- Group 1: Animals received dorsal root transection followed by transplantation of primary cultured mucosal OECs (mOECs) delivered within a collagen gel matrix (*n* = 10). This group functioned as a positive control, based on prior evidence demonstrating the capacity of mOECs to support functional recovery [23].
- Group 2: Animals received dorsal root transection followed by transplantation of cryopreserved mucosal OECs (CmOECs) embedded in collagen gel (*n* = 13).
- Group 3: Animals received dorsal root transection followed by transplantation of cryopreserved mucosal OECs (CmOECs) in collagen gel and were euthanized at 1-week post-transplantation to assess short-term survival of the transplanted cells (*n* = 2).

### 2.2. Cell Preparation

Donor animals were euthanized by gradual exposure to increasing concentrations of carbon dioxide (CO_2_), after which the heads were removed under aseptic conditions. The nasal septum was accessed, and olfactory mucosa (OM) tissue was identified and carefully excised bilaterally under a dissecting microscope. Collected tissue was immediately placed in ice-cold DMEM/F12 (Gibco, Paisley, UK) supplemented with 1% penicillin–streptomycin (P/S; 10,000 U/mL penicillin, 10,000 µg/mL streptomycin; Gibco, Paisley, UK) and subsequently rinsed in Hanks’ Balanced Salt Solution (HBSS; Gibco, Paisley, UK) containing 1% P/S to remove residual mucus.

The OM tissue was transferred to a 35 mm culture dish (Thermo Fisher Scientific, Waltham, MA, USA) containing 2 mL DMEM/F12 and minced into ~1 mm^2^ pieces. Enzymatic digestion was performed using DMEM/F12 supplemented with 25 mg/mL collagenase type I (Sigma-Aldrich, St. Louis, MO, USA) at 37 °C for 45 min (min). Digestion was stopped by the addition of HBSS, and the tissue was mechanically dissociated through gentle trituration. The resulting suspension was passed through a 40 µm cell strainer into a 50 mL Falcon tube to obtain a single-cell suspension. Cells were pelleted by centrifugation at 300× *g* for 5 min, the supernatant discarded, and the pellet resuspended in fresh culture medium.

Cell numbers were determined using an automated cell counter (Countess C10228; Thermo Fisher Scientific, Carlsbad, CA, USA). Cells were plated at a density of approximately 2.6 × 10^6^ cells per 35 mm dish pre-coated with poly-L-lysine (PLL; Sigma-Aldrich, St. Louis, MO, USA). Cultures were maintained for 21 days, with medium refreshed every other day. The growth medium consisted of DMEM/F12 supplemented with 10% fetal bovine serum (FBS), 1% insulin–transferrin–selenium (ITS), 10 µM forskolin, 1% N-2 supplement, and 1% P/S (all from Gibco, Paisley, UK unless otherwise stated).

For transplantation studies, a separate cohort of cells was used. These were obtained from a collaborating laboratory as olfactory mucosal tissue derived from transgenic “green” rats. The tissue was processed in our laboratory using the same isolation and culture procedures described above. These cells were subsequently cryopreserved, thawed, and incorporated into collagen matrices prior to transplantation.

### 2.3. Cell Cryopreservation Procedure

After 21 days in vitro, cultures were rinsed with HBSS and incubated with 0.25% trypsin/EDTA (Gibco, Paisley, UK) at 37 °C for 15 min to detach the cells. Enzymatic activity was neutralized with complete culture medium, and cells were collected into Falcon tubes followed by centrifugation at 350× *g* for 5 min. The supernatant was removed, and the pellet was resuspended in freezing medium (Gibco, Grand Island, NY, USA).

Cells were aliquoted into cryogenic vials at a density of approximately 1 × 10^6^ cells/mL. Vials were placed in a controlled-rate freezing container (Mr. Frosty; Thermo Fisher Scientific, Waltham, MA, USA) and cooled gradually to −80 °C overnight before being transferred to liquid nitrogen for long-term storage.

After four weeks, cryopreserved samples were rapidly thawed at 37 °C. The suspension was centrifuged at 350× *g* for 5 min, the supernatant discarded, and the pellet resuspended in fresh culture medium. Cells were replated into 35 mm dishes at a density of ~1 × 10^6^ cells/mL and maintained for an additional 3 days prior to encapsulation.

### 2.4. Encapsulation of OECs in Collagen

Encapsulation was performed as previously described [16]. Briefly, cells were detached using 0.25% trypsin/EDTA (Gibco, Paisley, UK) and resuspended in DMEM/F12 supplemented with 10% FBS to terminate enzymatic digestion. Following trituration to obtain a single-cell suspension, cells were combined with type I rat tail collagen (SLS, Nottingham, UK), 10× Modified Eagle’s Medium (MEM; Sigma-Aldrich, St. Louis, MO, USA), 1 M sodium hydroxide (NaOH, Fisher Chemicals, Loughborough, UK), and deionised water (Gibco, Paisley, UK) to achieve neutralization.

A 250 µL aliquot of the cell–collagen mixture was dispensed into a 35 mm culture dish and allowed to polymerise, resulting in a gel with a final collagen concentration of approximately 4.8 mg/mL. Once set, gels were submerged in culture medium, which was replaced every other day. Each gel contained approximately 2 × 10^5^ cells and measured ~1 cm in diameter and ~1 mm in thickness.

Encapsulated cells were maintained in vitro for a further 2–3 days before being cut into ~2 mm^2^ pieces for transplantation.

### 2.5. Surgical Procedure

Unilateral transection of four dorsal roots was performed as previously described [16]. Surgical procedures were carried out under isoflurane anesthesia with peri-operative analgesia (buprenorphine; 0.05–0.1 mg/kg; Ceva Santé Animale, Libourne, France). A midline dorsal skin incision was made, and the T2 vertebral landmark was used to orient the surgical field. Hemilaminectomies were performed spanning C5 to T2 to expose the underlying spinal cord. The dura mater was carefully opened with microsurgical scissors to visualize the dorsal roots. The C6–T1 dorsal roots were then transected as close as possible to their entry zone into the spinal cord, ensuring a perpendicular cut relative to the cord surface. The severed roots were repositioned and stabilized using fibrin sealant (Tisseel fibrin sealant Kit; Baxter Healthcare, Thetford, UK).

Dorsal Root Transection with Primary Cultured mOECs Encapsulated in Collagen Gels: Animals in this group underwent the same surgical approach as described above. Following root transection, collagen-embedded primary cultured mucosal OECs (mOECs) were placed between the proximal ends of the cut roots and their original entry points at the spinal cord. The graft was secured in position using fibrin glue.

Dorsal Root Transection with Cryopreserved mOECs Encapsulated in Collagen Gels: The procedure was identical to that used for the primary cell transplant group, with the exception that cryopreserved mucosal OECs (CmOECs) were used. The collagen constructs containing CmOECs were positioned at the interface between the transected roots and the spinal cord and fixed in place with fibrin sealant.

Post-Operative Care: At the end of surgery, muscle and skin layers were closed using absorbable sutures (Vicryl Plus; Ethicon, supplied by VWR International Ltd., Lutterworth, UK). Animals were allowed to recover in a temperature-controlled environment before being returned to their home cages. A softened diet was provided post-operatively, and analgesia (buprenorphine, 0.05 mg/kg, subcutaneous) was administered once daily for three days.

To minimize self-mutilation (autotomy), a bitter-tasting deterrent paste (Henry Schein, Melville, NY, USA) was applied daily to the ipsilateral forepaw for the first two weeks. Autotomy occurred in two animals (one control, one transplant). Humane endpoints were predefined: animals exhibiting severe autotomy (involving more than two joints in a single digit or extending beyond the nail in two digits) were euthanized in accordance with project license guidelines.

Animals were housed under standard conditions (20–22 °C; 12-h light/dark cycle) with unrestricted access to food and water.

### 2.6. Functional Test and Analysis

Functional outcomes were evaluated using previously established behavioral assays [14,16], focusing on two primary measures: grasping error percentage and forepaw fault score. Animals were trained and assessed prior to surgery to establish an individual baseline of normal performance and to exclude animals that did not reliably complete the task. For testing, rats were placed on the lower rungs of a 1 m vertical grid inclined at 15° and allowed to climb to the top. Assessments were performed twice weekly, beginning one week before surgery and continuing for up to six weeks post-operatively.

Grasping performance was quantified as the percentage of failed grasping attempts during a full climb. A score of 0% indicated that all rungs were successfully grasped, whereas 100% reflected a complete inability to grip any rung. Partial errors were categorized on a graded scale from 1 to 4 based on the extent of forelimb displacement through the grid: Grade 1 corresponded to the paw reaching the level of the rung without achieving a grip; Grade 2 indicated extension to the wrist; Grade 3 to the elbow; and Grade 4 to the axillary region.

Each assessment consisted of three consecutive climbing trials per animal, which were video recorded and subsequently analyzed by two independent observers blinded to treatment allocation. Animals that developed autotomy were excluded from behavioral analysis.

### 2.7. Immunochemistry

For in Vitro study: Following thawing, cells were maintained in culture for 48 h prior to fixation in 4% paraformaldehyde (PFA; Sigma-Aldrich, St. Louis, MO, USA) prepared in 0.1 M phosphate buffer for 30 min. Cells were then rinsed in 0.01 M phosphate-buffered saline (PBS, Sigma-Aldrich, St. Louis, MO, USA) and incubated in a blocking/permeabilization solution containing 2% skimmed milk (Oxoid, LP6031, Thermo Fisher Scientific, Basingstoke, UK) and 1% Triton X-100 (Fisher Bioreagents, BP151-500, Thermo Fisher Scientific, Waltham, MA, USA) for 1 h at room temperature.

Primary antibody incubation was carried out overnight at 4 °C using a combination of mouse anti-p75 neurotrophin receptor (1:500; Millipore, Temecula, CA, USA) and rabbit anti-fibronectin (1:1000; Dako, Agilent Technologies, Glostrup, Denmark). After PBS washes, cells were incubated for 1 h at room temperature with species-appropriate fluorescent secondary antibodies (goat anti-mouse Alexa Fluor 488 and goat anti-rabbit Alexa Fluor 546, both 1:500; Molecular Probes, Invitrogen, Thermo Fisher Scientific, Eugene, OR, USA). Following further washes, samples were mounted using ProLong Gold Antifade Mountant with DAPI (Thermo Fisher Scientific, Waltham, MA, USA), which provides nuclear counterstaining as part of the mounting medium.

For quantitative analysis, each culture dish was subdivided into four quadrants. Within each quadrant, four non-overlapping fields of view (1.02 mm × 0.76 mm) were imaged across three fluorescence channels (red, green, and blue) using a Nikon Eclipse 55i microscope (Nikon Instruments Inc., Tokyo, Japan) at 100× magnification. Total cell counts, as well as the proportions of OECs and olfactory nerve fibroblasts (ONFs), were determined using Fiji (ImageJ-win64, version 1.54p; National Institutes of Health, Bethesda, MD, USA).

For in Vivo study: At six weeks post-surgery, animals were euthanised via CO_2_ overdose and transcardially perfused with 0.01 M PBS, followed by approximately 500 mL of 4% PFA. The vertebral column, spanning from the craniocervical junction to the upper thoracic region, was dissected and post-fixed in the same fixative for 2–3 days at 4 °C. Subsequently, the spinal cord and associated dorsal roots were carefully isolated under a dissecting microscope, preserving continuity between the roots, transplant site, and spinal cord.

Tissue samples were cryoprotected in ascending sucrose solutions (10% followed by 20%) until fully equilibrated. Serial horizontal sections (16 µm thick) spanning the C6–T1 levels were cut using a Leica CM3050 cryostat (Leica Microsystems, Wetzlar, Germany) and thaw-mounted onto slides. Sections were post-fixed in PFA for 30 min, rinsed in PBS, and blocked in 2% milk solution.

Primary antibodies were applied overnight at 4 °C and included mouse anti-pan-neurofilament (1:500; BioLegend, San Diego, CA, USA), rabbit anti-glial fibrillary acidic protein (GFAP; 1:500; Dako, Agilent Technologies, Glostrup, Denmark), and rabbit anti-green fluorescent protein (GFP; 1:500; Thermo Fisher Scientific, Waltham, MA, USA).

After three washes in PBS (15 min each), sections were incubated with appropriate Alexa Fluor-conjugated secondary antibodies (goat anti-mouse or goat anti-rabbit, 1:500, Thermo Fisher Scientific, Waltham, MA, USA) for 1 h at room temperature. Sections were then washed, mounted using ProLong Gold Antifade Mountant with DAPI, and coverslipped.

Fluorescent imaging was performed using a Leica TCS SP1 confocal microscope (Leica Microsystems, Wetzlar, Germany). Acquisition settings, including laser power, pinhole diameter, and detector gain, were adjusted to minimize spectral overlap and prevent signal bleed-through between channels.

### 2.8. Statistical Analysis

Data are presented as mean ± standard error of the mean (SEM). Statistical differences between groups were evaluated using either an unpaired t-test or Tukey’s post hoc multiple comparisons test, as appropriate. Analyses were performed using GraphPad Prism 10. Sample sizes are specified in the Materials and Methods, Results, and corresponding figure legends.

## 3. Results

### 3.1. The Proportion of OECs and Olfactory Nerve Fibroblasts (ONFs) in the Total Population

**Figure 1:**
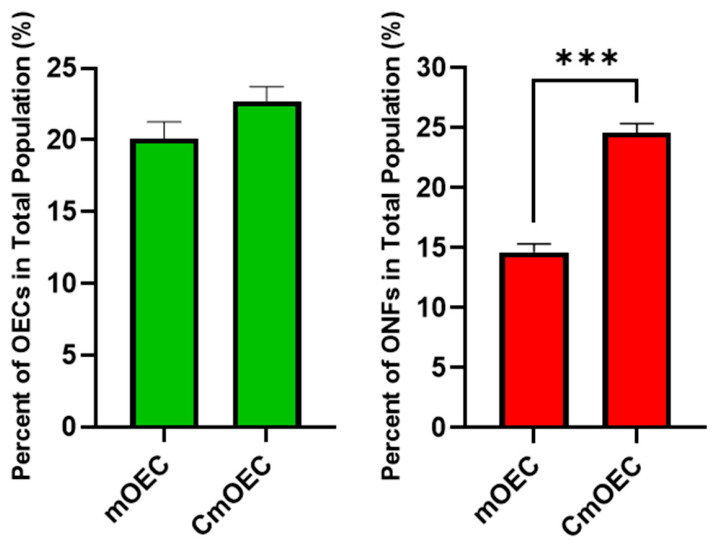
Proportions of olfactory ensheathing cells (OECs; green) and olfactory nerve fibroblasts (ONFs; red) within the total cell population in primary cultures (mOEC, *n* = 6) and cryopreserved cultures (CmOEC, *n* = 10). Data are presented as mean values, with error bars indicating ± SEM. Statistical comparisons were performed using an unpaired t-test, with significance defined as *** *p* < 0.001.

To assess the impact of cryopreservation on the proportions of olfactory ensheathing cells (OECs) and olfactory nerve fibroblasts (ONFs) in culture, cell populations were quantified using double immunostaining for p75 (OEC marker) and fibronectin (ONF marker). In primary cultures (*n* = 6), OECs made up approximately 20% of the total cell population, compared to around 23% in cryopreserved cultures (*n* = 10); this slight increase was not statistically significant. In contrast, the proportion of ONFs increased significantly, from 15% in primary cultures to 25% following cryopreservation (Figure 1).

### 3.2. Total Cell Population

**Figure 2:**
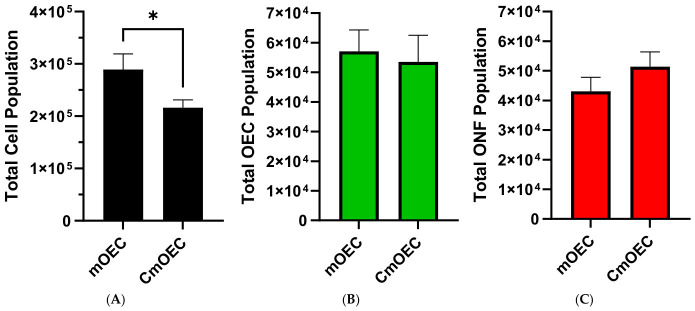
(**A**) Total number of cells per culture dish, (**B**) total OEC count per dish, and (**C**) total ONF count per dish in primary cultures (mOEC, *n* = 6) and cryopreserved cultures (CmOEC, *n* = 10). Data are shown as mean values, with error bars representing ± SEM. Statistical analysis was performed using an unpaired t-test, with significance set at * *p* < 0.05.

Analysis of the total cell population showed that primary mOEC cultures had a significantly higher overall cell count compared to cryopreserved mOEC cultures (Figure 2A). Despite this difference, the relative proportions of OECs and ONFs remained similar between the two groups, as illustrated in Figure 2B,C.

**Figure 3:**
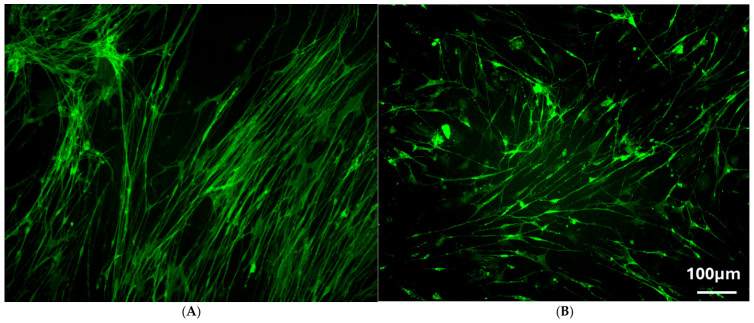
Images of immunostaining of p75 (Green), (**A**) Representative images of immunostaining of p75 to show normal primary mOEC culture after 21 days. (**B**) Cryopreserved mOECs after thawing from liquid nitrogen and cultured for an additional 3 days. Scale bar: 100 µm.

To evaluate the effects of cryopreservation on cell morphology, fluorescent micrographs were captured and analyzed. As shown in Figure 3A, mucosal OECs cultured for 21 days displayed a characteristic spindle-shaped morphology, featuring small cell bodies, narrow cytoplasm, and long, thin bipolar processes. These cells were directionally organized, forming aligned rows. In contrast, Figure 3B shows mucosal OECs after cryopreservation and an additional three days in culture. Although the overall morphology was similar to that of the primary culture, the cells appeared less organized, with processes extending in multiple directions rather than aligning uniformly with neighboring cells.

### 3.3. Functional Analysis

Prior to surgery, all animals underwent training to climb the cage to establish baseline pre-injury grasping error and forepaw fault scores. At this stage, all animals demonstrated a 0% Grasping Error Percentage and a Forepaw Fault Score of 0.

**Figure 4:**
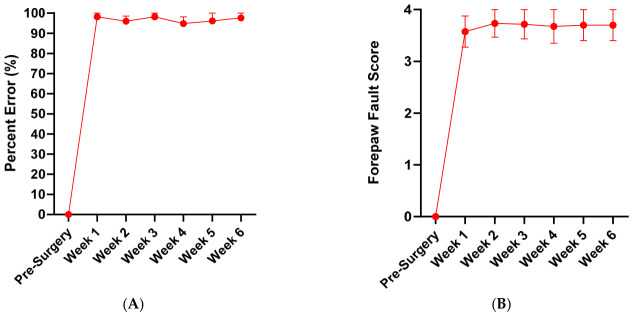
Grasping error percentage and forepaw fault score in the control group. (**A**) Percentage error and (**B**) forepaw fault score measured during the vertical climbing task across a 6-week period. Data are presented as mean values, with error bars indicating ± SEM.

Control group: In animals that underwent dorsal root transection alone (*n* = 6), grasping ability deteriorated significantly, with the percentage error rising to 100% by one week post-surgery, indicating a complete inability to perform successful grasping attempts. No recovery was observed over the 6-week period (Figure 4A). Similarly, forepaw fault scores increased to approximately 3.6 at one week post-injury, reflecting severe forelimb misplacement, with the limb slipping between the bars up to the elbow or axilla. This level of impairment persisted, with scores remaining around 3.7 throughout the duration of the study (Figure 4B). In our previous study, we also demonstrated that transplantation of collagen gel alone into the dorsal root injury site did not lead to any functional recovery [31].

**Figure 5:**
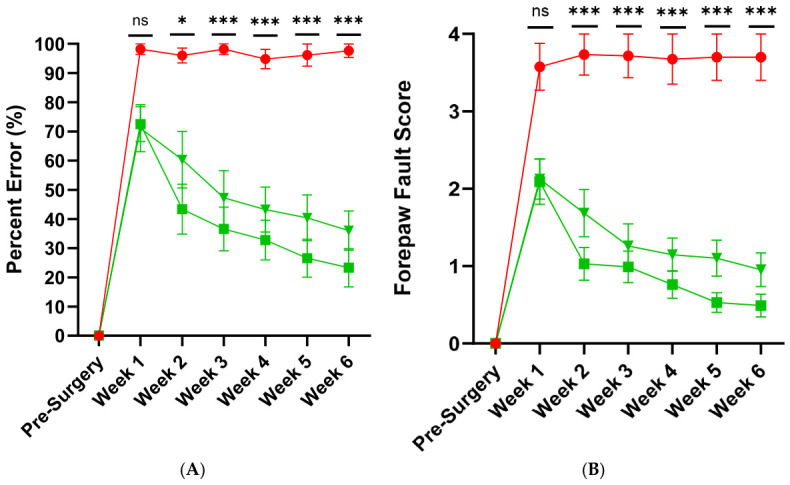
Comparison across experimental groups. (**A**) Percentage error and (**B**) forepaw fault score over a 6-week period. Groups included dorsal root transection only (red circles), mOEC transplantation (green squares), and CmOEC transplantation (green triangles). Data points represent group means, with error bars indicating ± standard error of the mean (SEM). Statistical analysis was performed using Tukey’s multiple comparisons test (ns = Not Significant, * *p* = 0.05, *** *p* < 0.001).

Transplant Groups: Animals that received dorsal root transection followed by transplantation with either primary cultured mOECs (*n* = 10) or cryopreserved mOECs (CmOECs; *n* = 13) encapsulated in collagen gel demonstrated significant functional improvement. In the mOEC-treated group, the initial climbing error rate was 73%, indicating partial recovery compared to the 100% error observed in the control group. Over the 6-week period, this error rate steadily declined to approximately 23%, representing a 77% overall improvement (Figure 5A). Similarly, the CmOEC-treated group showed an initial error rate of 71%, which decreased to around 36% by week 6. There were no statistically significant differences between the two transplant groups or between pre-surgical and 6-week post-surgical values within each group (Figure 5A).

Forepaw fault analysis supported these findings. Both treatment groups started with a score of approximately 2.0 (Figure 5B), reflecting partial forelimb function where the paw reached the grid but failed to grasp, or extended only to the wrist. This performance was already a marked improvement over the control group. By week 6, forepaw fault scores improved to 0.5 in the mOEC group and 1.0 in the CmOEC group, indicating enhanced grasping ability in both cases. Again, no significant difference was found between the two transplant groups or between pre- and post-surgical scores within each group (Figure 5B).

Both mOEC and CmOEC transplant groups showed statistically significant reductions in climbing error percentage and forepaw fault scores when compared to the dorsal root transection-only group (Figure 5A,B).

### 3.4. Immunohistochemistry

**Figure 6:**
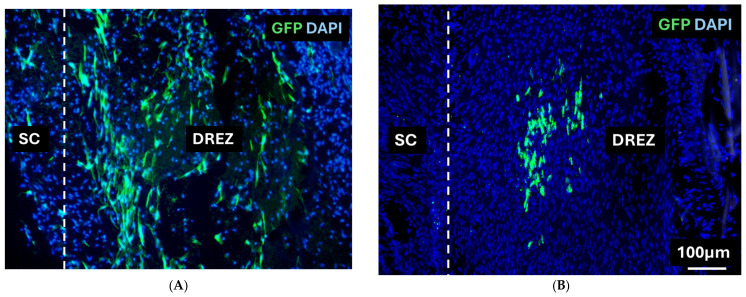
Immunostaining of GFP (Green) and counterstained with DAPI (Blue) in the transplant groups at (**A**) 1 week post-transplantation and (**B**) 6 weeks post-transplantation. SC, spinal cord; DREZ, dorsal root entry zone.

Short-term and long-term survival of transplanted cells: Transplanted CmOECs were detected by green fluorescent protein (GFP) staining at both one and six weeks post-transplantation. At each time point, GFP-positive cells were present at the dorsal root entry zone (DREZ) (Figure 6A,B), having migrated out of the collagen gel and integrated into the host tissue at the injury site. The transplanted cells displayed elongated processes oriented parallel to the host dorsal root and dorsal column axons (Figure 6A,B). A greater number of GFP-positive cells were observed at one week compared to six weeks post-transplantation.

**Figure 7:**
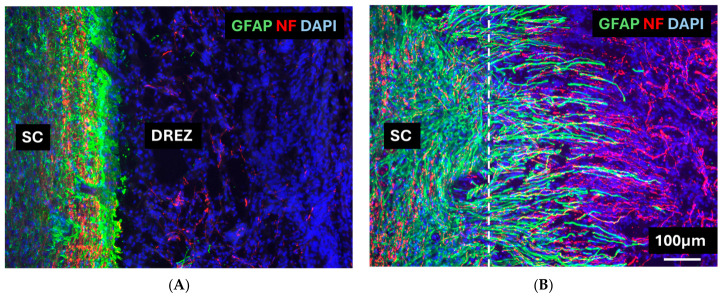
Images of horizontal sections immunostained for GFAP (Green), NF (Red), and counterstaining with DAPI (Blue) in (**A**) the Control group and (**B**) the CmOEC transplant group at the DREZ (dotted line) at 6 weeks post-transplantation. SC, spinal cord; DREZ, dorsal root entry zone.

Responses of the host astrocyte at the dorsal root entry zone, Control groups: Sections were double-immunostained for neurofilament (NF) and glial fibrillary acidic protein (GFAP). In the dorsal root entry zone (DREZ) of control animals, no NF-positive axons were detected (Figure 7A), and dense GFAP-positive astrocytic processes formed a barrier at the DREZ (Figure 7A). This contrasts with the pronounced axonal presence and astrocytic process extension seen in the cell-transplanted groups (Figure 7B, see below).

Transplant group: Double immunofluorescence staining for GFAP (green) and NF (red) revealed distinct differences in the organization of the DREZ between transplant and control groups. In the transplant group, NF-positive axons extended from the spinal cord into the DREZ, while GFAP-positive astrocytic processes projected from the spinal cord into the transplant region. These astrocytic processes were closely associated with and interwoven among the NF-positive axons. This configuration, consistent with our previous findings using bOECs and modified mOECs, formed a structural bridge between the severed dorsal roots and the spinal cord, potentially enabling regenerating axons to enter the CNS (Figure 7B).

## 4. Discussion

The present study demonstrated that cryopreserved mucosa-derived OECs (CmOECs) maintain the capacity to survive, integrate, and promote recovery following dorsal root injury, with behavioral and histological outcomes comparable to those achieved using primary cultured mOECs.

Mucosal OECs offer notable advantages over bulb-derived cells in terms of clinical feasibility. Unlike the olfactory bulb OECs, which require intracranial surgery to collect, olfactory mucosa can be obtained via a minimally invasive nasal biopsy under local anesthesia. In this study, we demonstrated that CmOECs remain effective in promoting functional recovery following dorsal root injury in rats, producing outcomes comparable to those observed with primary mucosal OECs (mOECs). This finding is particularly important because mOEC biopsies typically yield fewer cells per sample than bOECs [32]; therefore, cryopreservation would allow for multiple autologous or allogeneic samples to be pooled to achieve sufficient cell numbers. Furthermore, it facilitates the establishment of OEC biobanks, which could eliminate delays between cell collection and transplantation, potentially reducing the impact of secondary injury [24,25,26] and providing an on-demand cell source suitable for both acute and chronic injuries.

In Vitro Effects of Cryopreservation

Our in vitro study shows that cryopreservation markedly decreased the total number of viable cells in culture (Figure 2A), consistent with previous studies that attribute cell loss to osmotic and oxidative stress induced by freeze–thaw cycles [33,34]. Despite this reduction, the proportion of OECs remained relatively stable, at 20% in primary cultures versus 23% in cryopreserved cultures, suggesting that mOECs are relatively resilient to cryopreservation stress (Figure 1). This contrasts with our previous findings on CbOECs, which exhibited reductions in both OEC and ONF populations [31]. The preservation of mucosal OECs may reflect either intrinsic cellular resilience or enhanced protection from media supplements, such as ITS, N2, or forskolin (Fsk), that are present in the mucosal media but not the bulb. The observed increase in ONF survival in CmOEC versus CbOEC cultures further suggests that these supplements may support proliferation or cryoprotection. Indeed, some studies indicate that Fsk can improve cell survival during cryopreservation [35,36]. Further studies comparing cryoprotectants and media components may clarify the mechanisms underlying this difference in cell survival.

Morphological analysis further confirmed that cryopreserved OECs largely retain their phenotype. Both primary and cryopreserved OECs exhibited the spindle-shaped morphology with elongated processes (Figure 3A,B). However, CmOECs appeared less organized, with reduced alignment between neighboring cells, which may reflect altered intercellular signaling or polarity after cryopreservation, or simply shorter culture duration compared with the primary cells. Importantly, these morphological differences did not appear to impair functional properties, as demonstrated by the in vivo outcomes.

Our in vivo results confirmed the regenerative efficacy of CmOECs. Animals receiving CmOECs or primary mOECs transplanted into the dorsal root injury site exhibited significantly improved performance in both the vertical climbing and forepaw fault tests compared to injury-only controls (Figure 5A,B). The mOEC-treated group reduced climbing errors from 73% to 23%, while the CmOEC-treated group improved from 71% to 36% over six weeks. Likewise, forepaw fault scores decreased from ~2.0 to 0.5 (mOEC) and 1.0 (CmOEC). These results align closely with our previous studies using cryopreserved bOECs [31], where comparable recovery was observed, and further support the conclusion that cryopreservation does not impair the therapeutic efficacy of OECs.

Immunostaining for GFP at one and six weeks post-transplantation revealed transplanted CmOECs located at the dorsal root entry zone (DREZ) (Figure 6A,B). This indicated that the cells had migrated from the collagen gel into the surrounding host tissue and displayed elongated processes aligned parallel to host dorsal root and dorsal column axons. The majority of GFP+ cells at both time points were situated within the transplant region, though fewer were visible at six weeks. This reduction may reflect migration away from the DREZ, downregulation of GFP expression, or gradual cell loss, which is a pattern consistent with many other studies [7,37].

Short-term survival is consistent with previous OEC transplantation studies and suggests that the beneficial effects of OECs may result not from long-term engraftment but from their early influence on the injury microenvironment [38]. OECs are known to secrete a range of neurotrophic factors, modulate inflammation, and facilitate axonal regeneration [39]. Their early presence may help re-establish a permissive environment for axonal growth, even if the cells themselves do not survive long term. This characteristic could be particularly advantageous for allogeneic transplantation, as it implies that patients may not require prolonged immunosuppression. Future studies would need to be conducted to determine the minimum survival period needed for OECs to induce lasting functional recovery, which would in turn define the necessary duration of immunosuppressive treatment.

Double immunofluorescence for GFAP and NF provided clear evidence of transplant-mediated changes in the DREZ. In control animals, no NF-positive axons were detected at the DREZ (Figure 7A), and no astrocytic processes were extending from the spinal cord into the lesion; instead, a dense GFAP-positive barrier was formed, consistent with the formation of a glial scar that restricts axonal entry. By contrast, in the CmOEC group, NF-positive axons penetrated into the DREZ, closely associated with GFAP-positive astrocytic processes projecting from the spinal cord into the transplant site (Figure 7B). These findings indicate that OEC transplantation induces significant remodeling of the DREZ microenvironment, facilitating axonal regeneration into the spinal cord. The close association between regenerating axons and astrocytic processes in the transplant group suggests that OECs modulate the astrocytic response, preventing scar formation and instead promoting a more permissive environment.

In the present study, transplantation was performed in an acute/subacute timeframe under tightly controlled experimental conditions to allow clear attribution of observed effects to the transplanted cells. While this approach provides a robust and well-defined experimental model, it does not fully capture the temporal complexity of clinical spinal cord and dorsal root injuries. Future work will build on these findings by investigating delayed transplantation paradigms (e.g., 1–2 weeks post-injury), which more closely reflect clinically relevant scenarios. Such studies will also enable the influence of the evolving injury environment, including scar formation, inflammation, and tissue remodeling, to be systematically explored in the context of OEC-mediated repair. This will be important for optimizing therapeutic timing and further defining the translational potential of OEC-based interventions.

## 5. Conclusions

This study supports the clinical development of cryopreserved mOECs as a viable alternative to primary cultures. Our findings demonstrate that cryopreserved olfactory mucosa-derived olfactory ensheathing cells (CmOECs) retain their capacity to survive, integrate, and promote functional recovery after dorsal root injury, with outcomes comparable to those of primary cultured mOECs. Although cryopreservation reduced overall cell yield, the proportion of OECs remained stable. Behavioral testing confirmed significant recovery in grasping and forepaw placement following CmOEC transplantation.

Cryopreservation enables the creation of standardized, quality-controlled OEC biobanks that can be stored long-term and rapidly deployed in both acute and chronic injury settings. This has major implications for future clinical applications, as it addresses key limitations associated with primary cell cultures, particularly the risk of insufficient cell yield or culture loss due to contamination. By establishing well-characterized, ready-to-use cell banks, cryopreservation reduces logistical constraints related to culture preparation, ensures greater consistency between clinical batches, and facilitates both autologous and allogeneic transplantation. This flexibility broadens the treatment window and enhances the practicality and scalability of OEC-based therapies.

## Data Availability

The original contributions presented in this study are included in the article material. Further inquiries can be directed to the corresponding author.

## Abbreviations

The following abbreviations are used in this manuscript:

bOEC	Olfactory Bulb OECs
CmOEC	Cryopreserved Olfactory Mucosal OECs
CO2	Carbon Dioxide
d.H2O	Deionized Water
DAPI	6-diamidino-2-phenylindole
DMEM/F12	Dulbecco’s Modified Eagle Medium F-12 nutrient mix
DREZ	Dorsal Root Entry Zone
FBS	Fetal Bovine Serum
Fsk	Forskolin
GFAP	Glial Fibrillary Acidic Protein
GFP	Green Fluorescent Protein
HBSS	Hanks’ Balanced Salt Solution
ITS	Insulin–Transferrin–Selenium
Min	Minutes
mOEC	Olfactory Mucosal OECs
NF	Neurofilament
OB	Olfactory Bulb
OEC	Olfactory Ensheathing Cells
OM	Olfactory Mucosa
ONF	Olfactory Nerve Fibroblasts
p/s	Penicillin/Streptomycin
PBS	Phosphate-Buffered Saline
PFA	Paraformaldehyde
PLL	Poly-L-lysine
SCI	Spinal Cord Injury
SD	Sprague–Dawley
